# Losing Ourselves: Active Inference, Depersonalization, and Meditation

**DOI:** 10.3389/fpsyg.2020.539726

**Published:** 2020-10-29

**Authors:** George Deane, Mark Miller, Sam Wilkinson

**Affiliations:** ^1^Department of Philosophy, University of Edinburgh, Edinburgh, United Kingdom; ^2^Department of Informatics, University of Sussex, Brighton, United Kingdom; ^3^Department of Sociology, Philosophy and Anthropology, University of Exeter, Exeter, United Kingdom

**Keywords:** active inference, depersonalization, meditation, error dynamics, self

## Abstract

Disruptions in the ordinary sense of selfhood underpin both pathological and “enlightened” states of consciousness. People suffering from depersonalization can experience the loss of a sense of self as devastating, often accompanied by intense feelings of alienation, fear, and hopelessness. However, for meditative contemplatives from various traditions, “selfless” experiences are highly sought after, being associated with enduring peace and joy. Little is understood about how these contrasting dysphoric and euphoric experiences should be conceptualized. In this paper, we propose a unified account of these selfless experiences within the active inference framework. Building on our recent active inference research, we propose an account of the experiences of selfhood as emerging from a temporally deep generative model. We go on to develop a view of the self as playing a central role in structuring ordinary experience by “tuning” agents to the counterfactually rich possibilities for action. Finally, we explore how depersonalization may result from an inferred loss of allostatic control and contrast this phenomenology with selfless experiences reported by meditation practitioners. We will show how, by beginning with a conception of self-modeling within an active inference framework, we have available to us a new way of conceptualizing the striking experiential similarities and important differences between these selfless experiences within a unifying theoretical framework. We will explore the implications for understanding and treating dissociative disorders, as well as elucidate both the therapeutic potential, and possible dangers, of meditation.

## Introduction

In daily life, we take for granted the existence of a self: we feel that we are possessors of certain qualities, the experiencers of certain sensations, that we are different and distinct from one another, and that we endure from day to day. And yet, these assumptions have long been the focus of skepticism within both Western and Eastern philosophical traditions. Thinkers from various disciplines (e.g., from philosophy of mind, cognitive science, phenomenology, and Buddhist philosophy) are beginning to collaborate on various topics revolving around self and subjectivity. One lens through which philosophers and cognitive scientists have been recently exploring the self is through cases where subjects report a loss, or diminishment, of their sense of self. These reports occur most prominently in the context of psychiatric disorders such as depersonalization (e.g., Colombetti and Ratcliffe, 2012; Seth et al., 2012; Miller et al., 2020), meditation (e.g., Britton, 2019; Lutz et al., 2019), and psychedelic drugs (e.g., Millière, 2017; Deane, 2020). The active inference framework—a popular approach to modeling action and perception that uses principles of variational Bayesian inference (Friston et al., 2017)—is particularly promising for understanding these phenomena.

Our aim in this paper is to provide an updated account of selfless experience within the active inference framework^1^. By selfless experience, here, we mean the diminished sense of self that is reported in a wide variety of cases including depersonalization and meditative insight. Active inference and predictive processing have already been used to provide accounts of depersonalization in psychiatric contexts (Seth et al., 2012; Gerrans, 2019), and although we find these accounts promising, we seek to build on them in important ways. In particular, we differ from existing accounts in taking affective valence and control to be central to the sense of self. Building on existing accounts of self-modeling within an active inference framework (Seth, 2014; Hohwy and Michael, 2017; Friston, 2018), our account casts the self-model in terms of an allostatic control model (ACM; Deane, 2020), which we unpack in terms of “agentive control” and “motivational” components. The central thesis of this view is that the self is understood as an inference about endogenous causes of self-evidencing outcomes. In simple terms, this could be understood as the system modeling what it *wants* (motivations) and what it *can do* (abilities). However, we do not simply adopt the ACM for its own sake—there are concrete explanatory payoffs. In particular, we are better able to account for the wide range of selfless experiences under a single unifying framework. Selfless experiences come in a variety of flavors, ranging from the dysphoric and dysfunctional experiences associated with depersonalization to the euphoric and potentially superfunctional states sought after by meditators. Our explanation of this difference is, as we will see, an intrinsic part of our account of the emergence of these phenomena themselves.

An important addition to this literature that we will make is a reinterpretation of the role that affect plays in these processes. We will argue that the sense of self arises from the system’s evaluation of its own performance, or predictive control, of its own adaptive behaviors. As we will see, the tracking of our performance, and the allocation of resources (i.e., setting of precision), is being done in part by affective systems. That is, we quite literally feel how well adapted we are to a situation, and those feelings move us in ways that are intended to improve that fit. This has the consequence that our sense of being a self and affect are mechanistically intertwined. This updated theoretical account of selfhood then allows us to propose a more unified framework for understanding various alterations in selfhood and affectivity.

We proceed as follows: In *From the Free Energy Principle to Hierarchical Predictive Processing*, we give an overview of the free energy principle and hierarchical predictive processing. In *A Control-Theoretic Perspective*, we position these frameworks within a control theoretic perspective and show how allostasis can be formalized in terms of active inference. In *Affect in Deep Self-Models*, we build on these ideas to present an account of self-modeling in terms of allostatic control. In *Active Inference Accounts of Depersonalization* and *ACM Account of Meditative Selflessness*, we apply this model of the self to address depersonalization and selfless experiences attained through meditation, respectively. We wrap up and conclude by comparing and contrasting these two dysphoric and euphoric selfless experiences.

## From the Free Energy Principle to Hierarchical Predictive Processing

The free energy principle (FEP; Friston, 2010) is an ambitious unifying and overarching theory of life, according to which biological systems naturally strive to minimize free energy.

The FEP starts from the observation of existence (Friston and Stephan, 2007; Friston et al., 2010) and seeks to understand how organisms maintain their existence by “tuning” to their environmental niche, where the quantity of free energy is understood as a measure of the disattunement (which is equivalent to model “uncertainty”) between the agent and environment (Bruineberg and Rietveld, 2014). Crucially, in order to exist and reproduce, agents must stay within conditions that are conducive to continued existence—such as avoiding an unacceptably high body temperature. Of course, this is *phenotype specific*—the conditions that make continued existence viable vary across species. Organisms must minimize free energy, which is equivalent to maximizing the evidence of their model and so their own existence (Friston, 2010, Hohwy, 2016). Maximizing model evidence in this way is called “self-evidencing” (Hohwy, 2016).

In animals like us (and many others), it has been proposed that free energy is minimized, at least in large part, by hierarchical predictive processing in the brain and central nervous system (Friston, 2005; Clark, 2013, 2015). What the brain has to do, on such a view, is minimize prediction error (free energy) as efficiently as possible. This requires it to come up with an overall hypothesis or model about what is going on in the world. This hierarchical model generates predictions, and if it is inaccurate, it generates *prediction error* and updates predictions accordingly.

A major challenge in model selection arises because the world is a noisy and ambiguous place. Thus, there exists, at any given time, more than one model that fits the incoming sensory signal. This is where the notion of *prior probability*, often shortened simply to *prior*, comes in (and with it, the Bayesian element of the framework). This is the background probability of the model independently of the evidence. For example, (adapting an example from Pezzulo, 2014) before I hear my downstairs front window creak open, there is a background probability concerning the likelihood that I might be burgled. Whether I live in a high- or low-crime neighborhood will influence the prior probability of the “that’s a burglar!” model in response to the sound of the creaking window (“the evidence”). Models are selected based on both fit with current evidence and their prior probability. This means that you can get trade-offs, for example, where a model with a relatively low fit has a sufficiently high prior probability to be selected. For example, in the case of the hollow mask illusion, the model with the best fit would be the (perceptually accurate) “hollow concave face” model, but the slightly lower fit “normal convex face” model has such a high prior probability that it is selected instead, giving rise to the illusion.

This captures what the brain has to do, namely, resolve ambiguity using priors (viz., in a Bayesian manner); however, it does not tell us how this is implemented physically in the brain. Put simply, the brain maximizes efficiency (minimizes free energy) by being proactive and anticipatory. In other words, the nervous system does not passively wait for inputs to come in. Rather, even at the earliest stages of sensory processing, inputs are greeted by a barrage of top–down prediction. This does not just save time; it also saves energy and bandwidth, since the parts of the incoming sensory signals that have already been accurately predicted do not need to be passed up the processing hierarchy. All that gets passed up is what is “newsworthy” (Hosoya et al., 2005), namely, prediction error. Putting this all together, the nervous system tries to minimize prediction error by coming up with successful hierarchical predictive models that are chosen in a Bayesian manner (namely, based on fit and prior).

There are two more important tweaks to this picture. The first is to do with second-order prediction dynamics, namely, how the brain deals with statistical volatility. This requires introducing the notion of *precision*. In short, the world that we live in does not just have variability but also predictable levels of variability. As a result, our nervous systems learn over time that there are contexts where environmental information is high quality (trustworthy) and other contexts where it is not. For example, in good lighting, visual information is relatively high quality, whereas in poor lighting, it is relatively low. What an optimal system will do in response to this is have a way of setting second-order precision, namely, of appropriately varying the extent to which prediction error should be taken seriously (adjusted as a function of the likelihood of prediction error being accurate or simply noise). In high-quality informational contexts, it is expected that predictions will be good, and so prediction errors will be given relatively high weight (or gain). In low-quality contexts, prediction errors will be taken less seriously. This turning up and down of the gain on prediction error signaling is most commonly called *precision weighting*, and it plays a role far beyond the second-order dynamics that we used to introduce it. It is central to attention (Hohwy, 2012), and to the bringing about of bodily movement, an issue to which we now turn.

The second tweak comes when we note that, for embodied creatures like ourselves, action is an ever-present part of our existence. The Bayesian picture just described makes it look like we are primarily in the business of updating our models to best fit inputs from the world. However, of course, there are two ways of responding to prediction error. You can, certainly, update the model to better fit the world, but you can also update the world to better fit the model. The former is known as *perceptual inferenc*e, and the latter is known as *active inference*. It is with the latter that you get a PP account of action and basic motivation more generally. Active inference, on our view, is central to allostasis, a notion we introduce shortly.

## A Control-Theoretic Perspective

The foundations of active inference can be traced to control theory. The idea that a system maintains existence by resisting environmental disorder by acting to remain within a limited repertoire of *phenotype-congruent* states is closely related to the notion of maintaining “essential variables” (Ashby, 2013), where an internal reference point (also known as a setpoint or goal signal) is compared to the current state and the system acts so as to restore conditions to the setpoint.

The principles of *control-oriented predictive regulation* (Seth and Tsakiris, 2018) are very similar.

Here, the brain applies the same inferential machinery of hierarchical predictive processing to infer and track key homeostatic variables, using prior expectations and afferent sensory information about the body coming “from within” (Craig, 2003). In order to stay alive, organisms have to execute the right actions to bring about state transitions that bring bodily states into reasonable bounds (Pezzulo et al., 2015). The phylogenetically endowed high precision on expectations for staying within homeostatically viable states means that the organism acts to realize prior beliefs corresponding to the maintenance of essential variables (“goal priors”), for example, eating to restore a blood sugar concentration to expected levels. While goal priors originate in the maintenance of essential variables (e.g., steady temperature, blood sugar levels, etc.), over the course of ontogeny, an organism can acquire new goal priors that are predictive on longer timescales of being relevant for maintaining homeostasis—such as staying within a particular social milieu (Matthews and Tye, 2019).

Active inference, then, formalizes homeostasis through a control theoretic lens. Homeostasis from this perspective is maintained not only through autonomic reflexes (i.e., sweating to cool down) but also by *prospective control*. Such systems anticipate future dyshomeostatic conditions before they arise and proactively act to avoid them. This prospective control relates to both inferences about current and future bodily states contingent on certain actions (Sterling, 2012; Seth, 2014; Pezzulo et al., 2015). This process of anticipatory action, by which the brain regulates the needs of the body, is known as *allostasis* (Corcoran et al., 2019). Active inference formally articulates allostasis, such that agents *anticipate* surprising outcomes before they arise and act in order to minimize uncertainty about potential future outcomes (Sterling, 2012; Pezzulo et al., 2015, 2018).

On the active inference formulation, the action selection process itself is cast as a problem of inference, where agents must infer the active sampling of the world that realizes prior preferences and minimizes uncertainty (Kaplan and Friston, 2018). Action selection, then, depends on the use of a deep temporal model, where policies (sequences of actions) are selected based on prior expectations of the quantity of free energy that the agent expects itself to average over time (“expected free energy”) *given* a particular policy or course of action (Pezzulo et al., 2015; Friston et al., 2017). Intuitively, some courses of action (such as riding in the train carriage) have lower expected free energy than others (such as riding on the roof). Crucially, this involves anticipating unfavorable or dyshomeostatic conditions before they arise and acting to minimize uncertainty about potential future outcomes (Friston et al., 2015, 2017). On this account, higher levels of the cortical hierarchy, tracking regularities unfolding on longer timescales (Kiebel et al., 2008), contextualize lower levels by anticipating the downstream consequences of action and selecting policies that minimize expected free energy according to these expectations (Friston, 2010; Pezzulo et al., 2015). For example, my longer-term goal of successfully catching the train includes expectations about what I need to do to get to the station on time, which in turn unpacks into subgoals such as getting in my car and lower-level action–prediction loops as I use the pedal, gearstick, and so on.

In order to minimize free energy over longer timescales, active inference requires balancing the *pragmatic* and *epistemic* value of different actions. The pragmatic (or instrumental) value of an action or action policy (a sequence of actions) refers to the probability of it resulting in sensory states that fulfill some prior preference or goal state, such as maintaining a viable body temperature. Epistemic value refers to the reduction in uncertainty or information gain expected under a given action or action policy (Kaplan and Friston, 2018). Epistemic action allows organisms to increase an agent’s ability to reduce free energy by increasing their understanding of the predictable aspects of the environment. Information-seeking behavior such as novelty seeking and curiosity can be accounted for within this formulation in terms of epistemic action (Friston et al., 2015; Mirza et al., 2016; Kiverstein et al., 2017; Kaplan and Friston, 2018; Pezzulo and Nolfi, 2019). Intrinsic motivation (and epistemic foraging) can be understood here in terms of uncertainty reduction (Barto, 2013). Simulations of economic decision making and epistemic foraging behavior have been built based on this view that the probability of a policy is proportional to expected free energy (Friston et al., 2014, 2015, 2017). Active inference formulations of planning and navigation have been used to dissolve the “explore–exploit” dilemma, as the agent simply needs to act so as to minimize uncertainty (i.e., free energy; Kaplan and Friston, 2018). Agents engaging active inference do not just keep themselves in the states that are expected; rather, they anticipate in order to minimize uncertainty about potential future outcomes (Schwartenbeck et al., 2013; Friston et al., 2014, 2015, 2017).

Now that we have introduced the control-theoretic notion of allostasis, and how it is achieved via active inference, we next go on to develop our view of the sense of self.

## The Sense of Self as a Model of Allostatic Control

This section will argue that the self is best understood in terms of an *allostatic control model* (ACM). Recently, a number of computational models of the minimal sense of self (namely, the self as implicitly present in everyday world-directed experience, rather than something more overt and explicit like the self-conception or narrative self) have been advanced in the active inference literature (Limanowski and Blankenburg, 2013; Seth, 2013; Apps and Tsakiris, 2014; Allen and Friston, 2016). Common to these proposals is that the sense of self arises inferentially within a hierarchical generative model. Our central claim is that the inferential self-model arises from the system tracking its own self-evidencing capabilities (Friston, 2018). The purpose of tracking these capacities is to infer confidence (precision) in potential action policies according to their expected free energy and thereby arbitrate between potential actions accordingly. Self-modeling of this kind, then, is fundamentally related to selecting allostatic or anticipatory actions, where the system preemptively infers and avoids unfavorable conditions before they arise. By casting the self-model in terms of allostatic control, we will connect our view in new ways to the prevalent theme in neuroscience about the rich relationship between affectivity and the self (Damasio, 2003; Seth, 2013; Allen and Tsakiris, 2018). This view can be understood formally in terms of a higher-level inference about “subjective fitness”—that is, a higher level of the generative model that scores the “fit” between the action model and the world (see Hesp et al., 2019 for a formal treatment and computational model of this idea). Conceptually, our view of the sense of self can be decomposed into “agentive control” and “motivational” components. We will present these in turn.

### Agentive Control

Recall that while perception involves updating the model to better predict the incoming sensory input, action changes the incoming sensory input to better fit the model. In selecting an action, then, the system implicitly infers itself as able to bring about the consequences of that action. A sense of agency, the sense of being the one in control of an action, naturally emerges here as part of model sampling (Friston et al., 2013)—in selecting an action, the system implicitly infers itself as able to bring about the sensory consequences of the action. On this view, the sense of control—the expectation of being able to bring about certain consequences given certain actions—is *learned* through past experiences of the system inferring its own agentive capacities.

This connects closely with preexisting accounts of the sense of agency, where the system infers its own agency based on the ability to predict the outcome of a given action (Haggard, 2017). Here, attribution to endogenous causes (self), as opposed to exogenous causes (world/other), occurs as the result of a “comparator model,” where the sensory consequences of an action are compared with the expected sensory consequences (Frith, 2014). This allows the system to sculpt and improve motor control, as the discrepancy between the sensory consequences of an action are compared with the predicted (intended) outcomes. The system can then act to iteratively reduce this discrepancy and refine motor commands (Miall and Wolpert, 1996; Wolpert and Flanagan, 2001).

Crucially for the current account, *control* is *temporally deep* (Pezzulo, 2018), such that the agent not only has predictions about the immediate consequences of actions but also of consequences extending into the future. The sensory consequences of a given action may be sensorially proximal (e.g., the immediate sensory consequences of hitting send on an email), or sensorially distal and abstract (e.g., the expectation that when you see that person they will know the information in the email). The system, then, must be able to track the outcomes of actions on multiple timescales. Within the generative model, lower and higher levels of the hierarchy track regularities unfolding at faster and slower timescales, respectively (Kiebel et al., 2008). For an organism with a temporally deep generative model, this includes tracking its expected control of actions on short timescales (e.g., the expected sensory consequences of taking a step) and using these inferences to inform inferences about the state of control on temporally deep timescales (e.g., being able to walk a distance). On this view, the system models itself as an agent according to this hierarchically deep inference about its own endogenous control of sensation via its actions. In other words, I have a sense of what I can do based on past experience of acting in the world and come to expect myself as a controller over my future actions.

### Motivation

The *motivational* component of our view of the self-model is understood in terms of *goal priors* and, as such, connects closely to views of selfhood grounded in interoception (Seth and Friston, 2016; Barrett, 2017; Seth and Tsakiris, 2018). This is because the system will be generally more concerned about controlling the “essential variables” (i.e., homeostatic set points like blood sugar levels) tracked by interoception than variables inferred through exteroception and proprioception, which are less likely to pertain directly to homeostasis (Seth, 2014; Seth and Tsakiris, 2018).

Creatures tracking longer timescales can augment this with *deep goal hierarchies* (see Pezzulo et al., 2018), where fulfillment of longer-term goals can be traded off with fulfillment of shorter-term goals. On this view, low-level maintenance of “essential variables” are phylogenetically endowed expectations that, due to an “*a priori* hyperprecision of visceral channels” (Allen and Friston, 2016, p. 7), the system must act to fulfill, rather than simply updating via perceptual inference. One example would be moving to the shade under a tree to maintain viable body temperature. Divergence from these fundamental, phenotype-congruent low-level prior expectations tunes attention and amplification of sensory signals. This manifests itself to the system as, for example, the feeling of hunger (interoceptive prediction error) or a violation of the “healthy body condition” prior in the case of pain (Ongaro and Kaptchuk, 2019). These interoceptive changes tune the organism to the appropriate action opportunities in the given context, such as finding food to resolve interoceptive prediction errors or removing the source of pain. Crucially, pain is tuned relative to expectations given the context (Moutoussis et al., 2014). In the case of an approaching bear, the prospective inference about imminent catastrophic prediction error of being eaten trumps the proximal pain of a twisted ankle, and the selected policy is running away. Put another way, hierarchically deep contextualization of interoceptive signals tunes an organism to appropriate actions and engagements with the environment (Pezzulo and Cisek, 2016) and assigns appropriate precision to priors and ascending prediction errors. For low-level drives and motivations, this is intuitive—the hungry organism is tuned to capitalize on eating opportunities present in the environment. Precision on goals tracking different timescales are continually being traded off between levels—such as refraining from eating chocolate cake in the present for the sake of a longer-term goal of sticking to a diet (Pezzulo et al., 2018).

The sense of self, then, emerges as the result of a hierarchically deep inference about the system’s control of its own self-evidencing outcomes. In the generative model, this means that the sense of self can be understood in terms of a higher-level inference about the “fit” between the current action model and the world. By fit here we mean how well or poorly one is doing at reducing error over time relative to expectations. As we will see in the next section, a key implication of this picture is that self-modeling is fundamentally affective, where affective changes in the body tracks how well the organism is doing at fulfilling its own goal priors (“subjective fitness;” Joffily and Coricelli, 2013; Seth and Friston, 2016; Kiverstein et al., 2017). An upshot of this picture is that self-modeling, and indeed the feeling of being a self, is connected to affect in ways previously underappreciated in the literature, as we will explore next.

### Affect in Deep Self-Models

The previous section argued for a view of self-modeling as a higher-level inference about the system’s allostatic control. In our view, as we will now see, minimal self-modeling and affect are coconstitutive, such that affect can be understood as an inference about the performance of the action model in bringing about self-evidencing outcomes. This section unpacks how inference about allostatic control, manifesting affectively, is central to the allocation of precision.

In tracking the performance or “fitness” of the model over time, the system becomes sensitive to *the rate of error reduction.* In selecting a policy, the system has prior expectations of the rate at which error is likely to be reduced over time. The system can then evaluate whether its performance at reducing error is better or worse relative to its prior expectations. We can think of each agent’s performance in reducing error then in terms of a slope that plots the various speeds that prediction errors are being accommodated relative to their expectations. Changes in the rate at which error is reduced (referred to as “error dynamics”) turns out to be an important source of information for a predictive organism, as it reflects the efficiency, and so the quality, of its action model performance over time. As such, error dynamics play an important role in tuning precision estimations—increasing or decreasing our beliefs in the reliability of the model generating the policy (Kiverstein et al., 2017; Hesp et al., 2019). If precision is set based on estimations of how likely some action is to lead to the expected result, then the efficiency—the rate at which error is reduced—of those actions to reduce error should be taken into consideration. Greater than expected error for a given policy is evidence that the system should downregulate precision on the action model. Sensitivity to error dynamics increases our capacity to reduce prediction error over longer timescales, as it affords a means to toggle confidence levels on the action model according to the volatility of the environment.

The phenomenological manifestation of this (subpersonal) sensitivity to error reduction rates over time is affect. There is a growing literature that supports the view that affective changes not only track changes in immediate divergences from the homeostatic ideal, as was the focus of earlier predictive accounts of interoception (see Seth, 2013), but also tracks the rate of change in error management over time (Joffily and Coricelli, 2013; Kiverstein et al., 2017; Van de Cruys, 2017). Valenced bodily feelings (i.e., positive and negative hedonic tone) are, in part, a reflection of how well or poorly we are reducing error over time relative to expectations. When error is being reduced slower than expected, and the organism is becoming increasingly disattuned to its environment, this change is marked by feelings of frustration and disappointment. The negatively valenced bodily feelings provide the organism with feedback about the reliability of the selected action policies, indicating a need to downregulate precision on those policies. In contrast, when error is being managed at a better than expected rate, the organism is gripping the scene well, the bodily feedback are positive feelings of hope and satisfaction, and precision is upregulated. It is intuitive that persistently worse than expected rates of error reduction on a given goal prior act as a disincentive to pursue that goal and motivate the system to select a more achievable goal, and doing well is motivating to continue to realize a certain goal. Precision does not just concern the organism here and now and its momentary state of uncertainty but is instead helping it to continuously improve working toward managing uncertainty over time. Importantly, positive and negative feelings alter precision relative to the rate at which we have come to expect errors to be resolved.

Affective valence here is being reimagined within the active inference framework as a domain general controller that tracks and assigns precision relative to changes in our expected rates of error reduction (that is, expected reductions in free energy; Kiverstein et al., 2017; Hesp et al., 2019). Inference about how well the system is self-evidencing as a whole is tracking a long-term dimension of the self, which is necessarily more invariant and abstract in virtue of tracking a longer timescale, showing less variability than “lower” aspects of the self-model that are more amenable to changing across contexts. Negative and positive feelings then track lesser than expected and greater than expected allostatic control, respectively. This higher-order inference about the system’s confidence in its own action model, used to modulate precision on expected free energy (Hesp et al., 2019), is a candidate computational correlate for the sense of self—the feeling of being an agent.

This account of self-modeling as mechanistically intertwined with affectivity is, at present, a theoretical proposal. However, recent work (most notably Hesp et al., 2019) provides proof of principle of how this theoretical framework can be modeled computationally. This is a very promising groundwork for future work in computational modeling that is able to tie both phenomenology and behavior to underlying computational mechanisms. An important consequence of highlighting this underappreciated link between affect and self-modeling in active inference is that it provides a bridge between these computational frameworks and the phenomenology of being a self (for another account of this, see Kiverstein et al., 2020). Bodily feelings here represent a prereflective source of information about how well an agent is doing in their predictive engagements. These feelings give them a sense of what they can do, of what is possible, and what is not possible (the sense of “I can”). We have a feel for what is possible in the world based on what we can do in the particular situation we find ourselves within. Above, we characterized bodily feelings as driving policy selection. The result is that one quite literally feels drawn to relevant action possibilities. These bodily feelings track which possibilities are relevant to an agent and move us to improve^2^. The result is an ongoing dynamic dialectic between agent and environment all circling around affectivity.

While the importance of this ongoing tension between bodily feeling and environmental affordances is easily overlooked when it is functioning well, alterations in this quality can have devastating effects on how one experiences oneself and one’s world. With the addition of these more recent computational models of valence as setting precision relative to changes in control, we have now for the first time at our disposal the means to provide the fullest expression of an active inference account of the sense of self. In the rest of this paper, we will use this more fully realized view of the self to propose a new unified account of the alterations in self-experience native to depersonalization and meditative insight.

## Active Inference Accounts of Depersonalization

Depersonalization disorder (DPD) is still a relatively neglected dissociative disorder. Dissociative disorders are a class of mental illness characterized by disruptions in perception, consciousness, and/or identity. These disruptions can cause various symptoms that are problematic for a person’s life including social relationships and work life. Recently, however, research into the phenomenon of depersonalization more generally is increasing in part due to the piqued interest of philosophers and cognitive scientists interested in the nature and function of the self. Depersonalization experiences potentially provide researchers with important glimpses into the neuropsychological mechanisms and functional profiles of our ordinary experiences of being a self (see Metzinger on philosophy and dissociative disorders). A hallmark of depersonalization is a disturbance in subjective experience. This commonly includes a sense of detachment or alienation toward themselves, their bodies, and their environments. While specific disturbances in self-related experiences (depersonalization) and their experience of the environment (derealization) can come apart, they commonly co-occur; we will have more to say about his co-occurrence shortly (Sierra and David, 2011).

London-based writer Gracie Lofthouse writes on her own experience of depersonalization in a recent article:

“The first time I can remember feeling like I didn’t exist, I was 15. I was sitting on a train and all of a sudden I felt like I’d been dropped into someone else’s body. My memories, experiences, and feelings—the things that make up my intrinsic sense of “me-ness”—projected across my mind like phantasmagoria, but I felt like they belonged to someone else. Like I was experiencing life in the third person” (Lofthouse, 2014).

Most people have some experience of this sort of state. If you have not, it can be difficult to understand, and indeed, sufferers of depersonalization commonly report difficulties in expressing their experiences (Simeon and Abugel, 2006, p. 80). Depersonalization symptoms can last for moments, or several years, and commonly accompany major depression, anxiety disorders, substance addiction, brain injury and disease, and emotional trauma. An increasingly popular view of depersonalization is that it may act like an “airbag” in traumatic situations: when fight or flight are unable to remove an overwhelming, emotionally painful, experience, then the affective system may have its volume turned down as a direct means of reducing the suffering. The result of this reduction is what Medford calls it “desomaticion” or “deaffectation” (Sierra et al., 2005; Simeon et al., 2008; Medford, 2012; Medford et al., 2016) and is potentially the cause of the characteristically strange phenomenon of losing something important about the self and the world (see, e.g., Sierra and Berrios, 1998; Radovic and Radovic, 2002; Medford et al., 2005; Sierra et al., 2005; Simeon and Abugel, 2006; Baker et al., 2007).

In our view, predictive processing has offered some of the most promising avenues for understanding depersonalization. Our main aim here is to build on these and to improve on them based on more recent developments in the literature on active inference and the view of the self-model outlined in the previous section. The main explanatory payoffs that we can see are not only that we can better explain depersonalization and related symptoms but that we are also well-placed to explain why some instances of loss of self can have a positive valence, while others do not. Ultimately, superficial similarities in what are described as experiences of “loss of self” mask deep underlying differences.

### Existing Predictive Processing Accounts of Depersonalization

Seth et al. (2012) were perhaps the first to apply predictive processing to depersonalization, and since then, Gerrans (2019) has also provided an account. Seth et al. (2012) build their account on the central notion of “conscious presence.” Since “presence” involves both a sense of oneself as present in the world, and the world as present to us, it casts depersonalization and derealization as two sides of the same coin. To briefly summarize their account, they build on work in schizophrenia research on the loss of the sense of agency (e.g., Frith, 1987; Blakemore et al., 2000) according to which this arises from imprecise predictions about the sensory consequences of actions (see also *Agentive Control*, above). This account gets adapted to account for presence. According to Seth et al. (2012),

“presence is the result of successful suppression by top-down predictions of informative interoceptive signals evoked (directly) by autonomic control signals and (indirectly) by bodily responses to afferent sensory signals. According to the model, disorders of presence (as in DPD) follow from pathologically imprecise interoceptive predictive signals.” (p. 2)

Our account builds on this in a number of respects. First, this account is based on a view of emotion as interoceptive inference. So is ours, in a sense, but what Seth and colleagues mean is emotion as interoceptive *perceptual* inference. In other words, as they explicitly state (p. 1), they are fleshing out the James–Lange theory of emotion (James, 1890) according to which emotion is perception of bodily (specifically visceral) change. Given a PP gloss, whereas perception is the result of model selection for minimizing prediction error from sense perception, emotion is simply model selection for minimizing prediction error from interoception. This is perceptual inference since the model has to accommodate the input. Building on our recent work (Miller and Clark, 2017; Wilkinson et al., 2019), we, in contrast, view emotion, and affect more generally, as involving *active* inference, too. In terms of ACM, it is a central part of allostasis. This brings us to another crucial difference with our view. The view of emotion as interoceptive model building tells us nothing about *valence*. And yet, emotion has valence: it tends to be either positive or negative (and to greater or lesser degrees). In contrast, we tie positive valence to allostatic control (and negative valence to lack of such control). This means, crucially, given what we say later, that valence falls naturally out of our account, both of affect in general but also of self-loss, both negative and positive, in particular.

Unlike Seth and colleagues’ account, Gerrans’ account does not take presence as a basic notion out of which both self and self-loss emerge for free. Instead, Gerrans appeals to the notion of a “self-model” or, perhaps more accurately, the idea that the self-features as part of an overall predictive model that determines conscious experience. Gerrans’ main point is that our ordinary experience of the world, and ourselves, is generated by a constant integration of cognitive, perceptual, and affective signals. Building on his earlier work with Chris Letheby (Letheby and Gerrans, 2017), the self here is part of a predictive model, one that works to explain away the affective changes that occur as the organism engages with its environment. When affective signals go missing, the predictive system needs to explain the absence.

Gerrans’ approach to explaining depersonalization focuses on the role of affect in the generation of our felt sense of presence (Seth, 2013). Like Seth et al. (2012), Gerrans concludes that when predictions about ordinary affective reactions are not fulfilled, the system generates the sense of the agent being no longer present in the experience. In short, Gerrans builds on Seth et al. but adds the self-more explicitly into the model. To the extent that Gerrans’ account is similar to Seth and colleagues’ account, it shares many of the same differences with our own. Nevertheless, we like the embellishment of adding the self as a feature of the predictive model. In a sense, we would agree with Gerrans that presence emerges from a basic notion of self rather than the other way around.

What both of these existing accounts have in common, with respect to depersonalization, is the focus on affective numbing based on alterations (viz., inaccuracies) to interoceptive predictive processing. We do not disagree. However, what we add to the picture is the idea that affect carries inbuilt valence, involves active inference (allostasis), and, crucially, plays a role in setting precision weighting. This has the welcome side effect of allowing us to neatly explain other features of depersonalization beyond simply affective numbing. It also generates an account of depersonalization according to which it is inherently a negative experience (rather than something that needs to be appraised as such after the fact). These two other accounts tell us why there might be a loss of sense of self but not why that is negative. Given the existence of extremely positive experiences of self-loss (“enlightened” states), the negativity of the experience is not something that should be taken for granted. Our explanation of this large difference in valence is that superficial similarities are masking quite radical differences in what is going on in the two cases.

In the next section, we will propose that ACM can do a better job at accounting for depersonalization experiences than previous PP accounts. In particular, we will develop a view of depersonalization as a loss of allostatic control.

### ACM Account of Depersonalization

Recall that the ACM casts the sense of self as underpinned by an inference about the system’s endogenous control of self-evidencing outcomes. The highest levels of the self-model are also the most enduring, due to their being the most invariant across contexts. These higher levels that track how well we are able to control our interactions with the environment (i.e., allostatic control), more generally, act as hyper-priors informing more domain general precision estimations. The result of the sense of self being hierarchically deep in this way is that a temporary loss of control within a particular context may not necessarily reduce a more general sense of control or a sense of control across contexts. In other words, someone can fail to play the violin well without losing confidence in their ability to live a good life.

This inference about allostatic control—manifesting as affective valence—plays a role in setting precision relative to changes in how well or poorly we are doing at reducing error given the context. Sensitivity to unexpected increases in prediction error rates—manifesting here phenomenologically as negative valence—acts as a disincentive to continue operating in a particular context (Kiverstein et al., 2017; Hesp et al., 2019). For example, when learning an instrument, if a certain song is too complex given our skill level, the feelings of frustration that arise could motivate task switching perhaps to a simpler song or to developing some of the skills necessary to eventually play the more complex tune. Task switching here offers a way for the system to get back to reducing error at a better rate.

However, what happens when the system cannot resolve the negative affect through task switching? In other words, how would such a system behave if unexpected error continued to rise regardless of perceptual updates and behavioral interventions? For example, in active inference terms, trauma could be understood as a massive influx of prediction error causing the system to drastically lower confidence (precision) in its action models (see Linson et al., 2020). In the case of physical trauma, the body’s integrity, which is highly expected, is seriously disrupted or damaged. The system in this situation is unable to reduce errors either by updating their models (perceptual inference) or acting in a way that will bring their expectations back in line with the current situation (active inference). This disparity between expected control of prediction error and the error-riddled reality produces huge amounts of negative affect, which, as we have discussed above, reduces certainty on the currently selected policy as a means of tuning the agent to better predictive opportunities.

If an external situation continues to create error (i.e., severe pain) over an extended period of time, and the agent cannot control the situation through switching domain or context (that would otherwise be controllable), the resulting drop in precision on expected free energy is going to be such that consequent transitions between higher level affective states will be forced into the same fearful state continuously. The ascending message from the negative “affective charge” (Hesp et al., 2019) will override the descending message from higher level policies (i.e., our ability to control error by task switching also fails to resolve the issue, and so we lose confidence domain general control). That means that you have exactly the same effect at the next level, whereby the desired state (positive affect) is never reached despite trying to control it, leading to a drop in the precision on expected free energy at that level, and so on upwards. Crucially though, for this to happen, the person would have to never give up the resistance to the fear/pain, i.e., maintain a high precision on that goal state (i.e., the phylogenetically expectation to have a healthy, well-functioning body). In time, the “hopeless” situation might eventually create a learned belief that, no matter what they do, they will always be in a negative valence state (i.e., valence state transitions are not conditioned by policies and are stable in “negative”). It would basically be a perfect storm for a gridlock situation where you have a strong preference against the negative state but then you also know that you cannot escape it no matter what you do. The only option is to dissolve the process that is creating the negative affect in the first place since nothing else can work. The consequence would be an unraveling of the process by which we form affective states (i.e., inferring confidence in expected free energy) and with it the sense of self ^3^.

The dampening of affect and the feeling of self-loss are intimately related here. In losing a sense of allostatic control, the system ceases to posit itself as a causally efficacious controller of sensations. Accordingly, in losing a sense that the system has allostatic control across contexts, the affective system ceases to tune to opportunities to reduce error. Nothing is motivationally salient because the system infers that it is not causally efficacious in bringing about self-evidencing outcomes, and as such, it infers a global loss of confidence in precision on action policies (see Kiverstein et al., 2020). The result would be that the world would lose some of its *phenomenal depth—*it would cease to solicit one’s engagements and so be perceived (just as DPD sufferers suggest) as two-dimensional, or flat (Medford et al., 2006, p. 93). The result is, as Colombetti and Ratcliffe write, that “The world ceases to matter, people and events are not salient anymore. With this, the world ceases to move and affect one through one’s body” (2012, p. 148; see also work by Sass and Parnas, 2003; Fuchs, 2005).

This proposal casts new light on various circumstances of occurrence surrounding experiences of depersonalization. For example, consider a traumatic stressor such as torture, which is perhaps the most reliable instigator of depersonalization (Kira et al., 2013). On the current account, sustained inefficiency of the motivational system (in this case, severe pain over an extended period of time) in a scenario where the person has no control to act to resolve the prediction error results in a global loss of confidence in “tuning” affective responses. Another example is major depression. Depression can be understood as a domain general inference of loss of allostatic control, where, in extreme cases, the system ceases to posit itself as a causally efficacious agent (Fabry, 2019; Kiverstein et al., 2020). The chronic stress of an uncertain or volatile environment means eventually that the system ceases to posit endogenous control on the outcomes as the occurrence of positive/negative outcomes is inferred to be independent of the agent’s actions. Through the current framework, the comorbidity of major depression with depersonalization can be understood to be a sustained loss of allostatic self-efficacy. Stephan and colleagues proposed that “the performance of interoceptive-allostatic circuitry is monitored by a metacognitive layer that updates beliefs about the brain’s capacity to successfully regulate bodily states” (Stephan et al., 2016, p. 1), which they dub “allostatic self-efficacy.” Other accounts have proposed that depression functions as a means of reducing prediction error associated with adverse social contexts (Badcock et al., 2017). If this is along the right lines, depression itself would function as a means of motivating withdrawal from potentially aversive contexts. The link between major depression and depersonalization can be understood here in terms of the system ceasing to posit itself as an endogenous controller of self-evidencing outcomes—when there are no possible context to move to (no high level, temporally deep goal priors to realize), the total loss of allostatic control is experienced as depersonalization^4^.

In the next section, we turn our attention to an example of a potentially euphoric selfless experience, namely, the sorts of selfless experiences that can arise from meditation. A view of the self-model in terms of allostatic control gives a fitting account of this and shows how this kind of selfless experience is radically different (indeed, relative to control, they are diametrically opposed) to selfless experience in depersonalization.

## ACM Account of Meditative Selflessness

While meditation is something of an umbrella term, the disciplined control of attention is central to almost all styles of meditation (Albahari, 2009; Austin, 2013; Garfield, 2015; Millière et al., 2018). For brevity, we will focus here specifically on *focused attention meditation*. Meditation here takes the form of consciously attending to a particular object (i.e., bodily sensation or breathing), and when the mind wanders from the chosen target and the practitioner realizes the shift, they actively “let go” of the distractor and reorient their attention back to the initial meditative target.

Active inference has recently begun to be applied to thinking about meditation (Farb et al., 2015; Pagnoni and Guareschi, 2015). Lutz et al. (2019) have provided the first account of *focused attention* meditation in terms of active inference. Focused attention meditation is described as having two interrelated aims: the pragmatic activity of regulating attention on a particular object (i.e., the breath sensations, an object) and the epistemological activity of increasing one’s understanding of the nature of the meditative object and the various distractors, in particular recognizing their dynamic and impersonal nature (Lutz, 28).

In active inference terms, the pragmatic aspect of focused attention meditation requires that top–down directed precision enhance the behavioral policies associated with stable attention on an object. The challenge for the meditator then is to maintain this policy, although multiple other policies may be simultaneously active and acting as competitors for selection (Pezzulo and Cisek, 2016). In meditation, this ongoing competition becomes simplified to include only the policy of maintaining attention on the meditation object and all other competing policies attempting to divert attentional resources elsewhere (e.g., spontaneous memories, future planning, homeostatic concerns, mind wandering, etc.). Inaction during this process is considered crucial, as it is the process of setting top–down precision on the sensory signals associated with the meditative object that allows this dialectic between focused attention and distraction to unfold and to be consciously attended to. Lutz and colleagues suggest that this quality of “inaction” corresponds to the subjective experience of “letting go” of the various distractions (p. 28).

Over time, the meditator can learn to allow the various distracting thoughts and sensations to arise and pass without disturbing their concentration, that is, without disrupting the meditation policy of focused attention. In part, this occurs through learning to actively reduce precision on the distracting (negative) goal prior. Inaction itself does this to some degree. An itch motivates, via negative valence, a scratching policy because of the preference for the non-itching state (i.e., goal prior) and the high precision on the itching state (i.e., resistance to the sensations), as well as the learned connection between the itch and the action policy scratching. By not acting, and calmly observing the itch, the negative preference loses precision (i.e., resistance to the sensations is dropped). The result is that the probability of the sensation driving the selection of a new (distracted) policy is also lessened. In other words, meditators can actively reduce certainty on goal states and mitigate involuntary policy selection through inaction. The result is that over time, such goal priors cease to draw processing (i.e., attention) in the same way.

In line with our view that the system infers its own control in terms of correspondence of action–outcome contingencies, here, the system learns endogenous control on the precision of goal priors through repeated reduction via opting for the focused attention policy. Over time, the decrease in distractibility can be understood as an increased ability to endogenously control precision on goal priors. The system here refines attentional selection through mental action in a way analogous to refining motor commands through iterative inference of control of action–outcome contingencies (Miall and Wolpert, 1996; Wolpert and Flanagan, 2001). Note that learning to actively adjust precision on goal priors in this way is to learn to exert exactly the kind of control that is missing or weakened in cases of depersonalization as characterized above. In those cases, an inability to reduce precision on a goal prior that was unattainable leads to tremendous suffering and a loss of confidence in our ability to control the world more generally. In learning that it can endogenously set precision, it now begins to infer a domain general sense of being able to realize its goal states. This increase in domain general control may correspond to the pervasive feelings of joy and peace that characterize long-term meditation (Dambrun and Ricard, 2011; Dambrun, 2016).

During the course of becoming an adept meditator, one develops the ability to remain poised between the object of attention and the ongoing flow of spontaneous mental activities. This capacity to remain subtly focused on the meditative object, while at the same time observing clearly the spontaneous mental activity, provides an optimal opportunity to learn about the process of policy selection taking place within its own system (Ridderinkhof et al., 2004). This is the epistemological element of focused meditation. The result of this introspective investigation is a gradual “opacification” of these mental processes (Carter et al., 2005). A mental process is considered transparent insofar as its contents are available to consciousness, while its non-intentional structure or construction process are not (Metzinger, 2003). Without having access to the earlier stages of processing, transparent processes are presented subjectively as fundamentally real and personally essential. Metzinger writes,

“Transparent phenomenal states make their representational content appear as irrevocably *real*, as something the existence of which you cannot doubt. Put more precisely, you may certainly be able cognitively to have doubts about its existence, but according to subjective experience this phenomenal content—the *awfulness* of pain, the fact that it is *your own* pain—is not something you can distance yourself from. The phenomenol-ogy of transparency is the phenomenology of direct realism and in the domain of self-representation it creates the phenomenology of identifi-cation.” (2017, p. 248).

By continually letting go, the distracting policy selections are observed as non-essential—they arise, they persist for some time, and they eventually dissolve without the need for overt actions. The system becomes aware of the constructed nature of these precision assignments (e.g., itching directly leading to scratching). Through repeated observation of this process, the system ceases to identify with the precision selections, exactly because it observes them to occur without attributing them to a “self” as an endogenous cause. Recall that the system infers itself as a self—as an endogenous cause of sensations—through agentive actions, where the correspondence between action–outcome contingency feels agentive. Through repeated reorientation of attention back to the meditation target (by reengaging the meditation policy), the automatic precision assignments (e.g., itch→scratch) cease to be identified as essential and so begin to lose the quality of immediateness and irrefutability that come with being transparent. Crucially, as the system observes these precision allocations occurring independently of agentive engagement (due to the non-action policy currently being engaged), the usual means of inferring itself as a self-due to the correspondence of action–outcome contingencies is disrupted, and processes occurring in the system increasingly appear as non-essential to the self. This point about opacification is an important one for our discussion about selfless experiences.

This ties in closely with the “non-self” themes in Buddhism. Common to all Buddhist schools is a critique of our ordinary self-experiences. The Buddhist doctrine of *no-self* (anattā) teaches that our ordinary self-experience is both mistaken and an important source of human suffering. The mistake is the common assumption that behind the various psychophysical processes that make up our conscious experiences, there is a single, essential subject, who is responsible for constructing and owning those processes. However, when we turn our attention inwards in an attempt to catch a glimpse of this assumed subject, all we ever experience is the dynamic and impersonal processes. This is the point—while we commonly assume there to be an essential and *unconstructed* subject, that sense of being a subject is in fact *constructed* from the interaction of our cognitive–behavioral processes. The Buddhist move here is not to deny that the sense of self is real, that quality of experience that delineates my sensations from yours. Rather, the selfless insight Buddhist meditators seek out is the transformative recognition that, while the self unreflectively appears to us as essential, persisting, and unified, it is in fact constructed, impermanent, and dynamic (see Davis and Thompson, 2017).

According to the Buddhist tradition, our mistaken assumptions about the self are generated and maintained by craving (*tanha* in Pali). Craving is a technical term here; it describes the felt urgency or motivational drive to make the world conform to our desires—our anxiety to perpetuate positive feelings and reduce negative ones. This ongoing emotional investment is what unifies the various impersonal psychophysical processes under a single idea: a persistent and essential self. In turn, the more we identify with our specific concerns or roles, the more intense the motivation to bring about those states in the world^5^. In humans, these desires expand beyond basic homeostatic concerns (i.e., being fed and watered) to include the wider constellation of ideas and roles we appropriate into our identity (i.e., being a student). In Buddhism, this craving is thought to be responsible for significant human suffering. Consider the difference in magnitude between the relatively short-lived pain of breaking an arm, and the potentially life long suffering of losing an opportunity to play a beloved sport professionally. At the heart of Buddhism is the teaching that, while pain is unavoidable (i.e., the broken bone), our reaction to it (i.e., the craving to be a professional player) is optional. It is the craving, and not the pain, that is thought to be transformed through meditation and with it the mistaken sense of self that craving engenders.

Meditation is presented as a vehicle for reducing craving and disrupting the mistaken view of self. In the satipatthana sutta, the foremost early Buddhist text on meditation, the student is directed to divide their experience into five categories (or “aggregates”) of phenomena: bodily form, valence, perception, volitional activities, and consciousness. These aggregates together are thought to create the experience of being a subject (Hamilton, 2000; Shulman, 2014). The aim of meditation here is to reduce one’s identification with these psychophysical processes by closely examining each individually and their interactions and systematically noting their impersonal nature. In Anattalakkhaṇa Suttam, the Buddha suggests that meditators observe each aggregate, saying to themselves “not-I” or “not-my-self.” As the various psychophysical processes that make up the self-come to be experienced as “not-I,” the constructed nature of the self becomes apparent.

In addition to non-meditation policies (i.e., distractions) being driven by changes in goal states, they are also driven by our affective reactions to those goal states. As we have seen, valence sets precision relative to our ability to reduce error given a certain goal. Ordinarily, once a goal state is selected (predicted), any hesitancy in responding in the ways that the system has learned to expect produces negative valence, which has the effect of increasing the drive on policy selection as an attempt to catch up to the predicted slope. As long as valence is experienced transparently, it has this powerful motivating effect on actions. The meditator then must also be able to reduce the certainty on the valenced reactions that occur from their commitment to non-action. By attending to the valenced sensations themselves (i.e., the discomfort of not reacting), the system learns that these signals too are changing and non-essential. As soon as one has made the observation that non-action makes them uncomfortable, these systems are already being rendered opaque. Focused attention meditation then simultaneously makes opaque precision on goal priors (i.e., the confidence in the degree of wanting or not wanting certain states) and precision on expected free energy reduction that is given affectively.

In Buddhism, reflecting on valence (*vedana* in pali) is considered especially important in the process of relinquishing craving and disrupting the mistaken view of the self. Valence is considered the “weak link” in the process that gives rise to both craving and the mistaken view of self (Anālayo, 2009). In non-meditators, valence conditions craving: pleasant feelings give rise to attachment; painful feelings give rise to resistance. In contrast, long-term meditators are thought to be able to experience valence without further craving-driven responses. The Buddha taught,

“Touched by that pleasant feeling he does not lust after pleasure or continue to lust after pleasure. That pleasant feeling of his ceases. With the cessation of the pleasant feeling, painful feeling arises. Touched by that painful feeling, he does not sorrow, grieve, and lament, he does not weep beating his breast and becomes distraught” (Ñānamoli and Bodhi, 1995, p. 334).

Notice that even for the “well-taught noble disciple” (that is, meditators who no longer operate under craving and illusory notions of the self), valenced states still arise. These states are in and of themselves neutral in terms of well-being (Harris, 2018). It is the strong motivational impulse to act (i.e., fleeing our pain; grasping at joys) in response to those signals that the Buddhist meditative project aims to transform. A close meditative investigation of valence is taught to have the effect of separating valence from craving, the result being that one begins to react with less preference relative to pleasure and pain. As Buddhist scholar Albahari writes, “As mental suffering is finally eliminated through insight [into the non-self-nature of these processes], unpleasant *vedanā* will be confined to only physical (not mental) suffering” (Albahari, 2014, p. 11). As our urgency to react in self-serving ways diminishes, so too does the illusion of being an enduring and essential subject. The culmination of this process of extinguishing craving is *nibbāna* [enlightenment]: “the final flash of insight that burns out *taṇhā* and the sense of self for good” (Albahari, 2014, p. 11).

The gradual opacification of valence results in various positive effects discussed in the Buddhist paradigm. As described above, inaction would allow for the opacification and dereification of the valence system. As valence is increasingly modeled (made opaque), it ceases to invoke that powerful sense of urgency (associated with the Buddhist notion of craving or taṇhaā*;* Albahari, 2014), which, as we saw above, results from valence being experienced transparently and so presented phenomenologically as both immediately real and essential to the self (Metzinger, 2003). This change is an important one, as it results in the loss of the driving force that perpetuates the sense that there is an essential subject within and behind the various processes (Albahari, 2014). As valence is made opaque, and control over goal prior precisions is achieved, craving ceases due to its disentanglement from the conditioning influence of pleasure or pain. Thus, the illusion of self is disrupted. In gaining endogenous control on the precision of goal priors, meditation therefore enables the system to pull these apart so that (dis)liking does not need to condition (not) wanting, allowing the suffering associated with craving (and aversion) to be avoided. Gaining insight into the process by which valence is driven by our goal priors would have the consequence of allowing one to actively separate the two darts discussed by the Buddha. One comes to understand how pain (deviation from a goal prior) leads to suffering (transparent negative valence signaling the deviation from the goal prior and its expected resolution); that suffering occurs only insofar as we desire to avoid the pain (high precision on the goal prior drives error dynamics); and finally, although we often cannot do anything about the pain itself, we can, by watching the whole process closely, render the valenced reactions opaque and so reduce the degree to which valence drives policy selection. That is, we can reduce the craving and suffering that arises from transparent valenced reactions simply by observing closely the link between pain and discomfort, thereby rendering the valence opaque.

The opacification of this part of the precision machinery opens new opportunities for control. Observing our valenced reactions allows us to develop new higher order policies about how precision (via valence) is being set on policies^6^. In other words, instead of valenced signals adjusting precision on policies directly, and so automatically conditioning us to behave in certain ways, one can now learn to activate alternative policies depending on the usefulness of the valenced signals. For example, mindfulness has been shown to be highly effective in helping people to quit smoking cigarettes (Bowen and Marlatt, 2009). The practice here is to disrupt the pattern leading from craving to using by selecting a policy to closely attend to the feelings of craving—the negative valence driving processing toward the expected rate of error reduction relative to nicotine levels—every time they arise. The new goal now activated every time there is a craving (instead of smoking a cigarette) is to watch, as closely as possible the arising, the progression and the inevitable depletion of the craving-related feelings. Overtime, these sorts of mindfulness practices have the effect of teaching the system that cravings are in fact just feelings in the body, which can be allowed to direct processing and behavior or not^7^. This discovery can represent a major return of control for people struggling with substance addiction. Notice that valence here does not go missing through this process of opacification (as it does in depersonalization). Rather, valence begins to be interpreted by the system as what it is: information that can be useful, but is not essential, in selecting policies.

## Conclusion

In this paper, we seek to better understand the nature of the self from the perspective of the increasingly popular active inference framework. Within this framework, we present a novel account of the self, in terms of an allostatic control model (ACM; Deane, 2020). Central to the ACM account is a view of affect as a second-order process that guides the predictive system (via precision weighting) toward opportunities to improve. This is a novel take on affect that we have been developing over a number of recent publications (Kiverstein et al., 2017, 2020; Miller et al., 2020).

We then put this account to work in trying to understand two starkly contrasting forms of self-loss, namely, depersonalization and the selfless experiences attained through meditation (viz., Buddhist no-self insights). Given our proposed framework, these two varieties of selfless experience are characterized by stark differences in the systems degree of control: whereas depersonalization is expressly characterized as resulting from a critical loss of inferred control, selflessness in the context of meditative practices is marked by a significant gain in control.

There is today, however, an increasingly popular idea that depersonalization and the selfless experiences attained through meditation are somehow closely related. Meditation teacher and neuroscience enthusiast Shinzen Young has called depersonalization the “evil twin” of the Buddhist notion of *enlightenment* (Lofthouse, 2014). Part of what motivates this association, so it seems, are similarities in first personal accounts of both kinds of selfless states. Sufferers of depersonalization and long-term meditators make surprisingly similar reports about reductions in their experience of being agents of their actions and as owners of their thoughts and behaviors. While these first personal accounts can sound very similar, given our framework, this is where the family resemblance ends.

As we have shown throughout this paper, there are important computational differences between these two selfless experiences. Of particular importance is the difference in how affective valence contributes to either state. Depersonalization is characterized as a loss of control, leading to the dampening of these affective valence systems. In meditation, there occurs a gradual opacification of the affective valence system. This opacification produces an important change in the meditator’s relationship with the positive and negative affect—specifically, by no longer being automatically appropriated into their self-model. This has the result that changes in valence no longer create the existential urgency for change they would otherwise. It is important to note here that valence remains perfectly intact, continuing to tune the agent toward opportunities to improve in their predictive success. The purpose of the practice is not to disrupt valence itself (as happens in depersonalization) but rather to become conscious of the precision estimators in a way that allows them to select more skillful and beneficial policies. What is permanently altered in the meditation process is the system’s reaction to those signals. While there is still the experience of frustration and joy, there is no longer the sense of being an essential subject to appropriate these states as “me” and “mine.” This insight leads to an eventual dissolving of our misguided idea that we are a single and enduring thing, to be replaced by an acknowledgment that we are a dynamic, self-organizing process. Far from reducing control, and in direct contrast to depersonalization, this development of one’s metacognitive abilities here allows one to contextualize and control precision estimations in new and powerful ways.

Notice then that for someone to transition from experiences of depersonalization to the selfless states attained through meditation, they would first need to regain their phenomenal access to those affective responses. Without affect playing its role in tuning the system (relative to its predictive success), the opacification and subsequent insight into the nature of those precision processes could not occur. To be clear, we are not suggesting that the endeavor of bringing affect back online for people suffering depersonalization should be carried out through meditation specifically^8^. Rather, our point is that those affective signals would have to first become available for introspective access in order to be modeled in the way facilitated by focused meditation. This follows from the fact that, on our account, it is not the loss of affectivity that results in the selfless experiences sought after by Buddhist meditators but the process of modeling those affective changes that opens the way for a new perspective of the self and a new layer of control to emerge.

In terms of control, depersonalization and selfless experiences sought after by mediators are, computationally speaking, polar opposites. And yet, there is something important to be said here about the potential focused attention meditation has to provoke depersonalization experiences. Britton’s *Dark Night Project* has documented and investigated a large number of personal reports on various difficulties that can accompany meditative practices^9^. Britton (2019) suggests that adopting a persistent attitude of turning toward difficult stimuli and focusing on negative emotions can lead to negative outcomes. This aspect of mindfulness training has positive effects for some people, primarily by helping them to facilitate a gradual sensitization to negative affect. Such exposure approaches to therapy are thought to work by reducing avoidance, which has been shown to play a leading role in the generation and maintenance of various psychological disorders (Barry et al., 2015). However, exposure therapies are most effective for people who tend toward high levels of avoidance (McNally, 2018). In other, low-avoidance personality types, anxiety and dissociative disorders can be produced and exacerbated by facilitating an attentional bias toward threat (MacLeod et al., 2002; Eldar et al., 2008). The fact is that the most effective treatment for an individual will depend on their baseline attitude toward threat. Given our account above, it makes sense why inappropriately meditating on traumatic events (that is beyond a certain healthy window of tolerance) could create depersonalization effects. If depersonalization is an airbag deployed when fight or flight would not work for getting one out of a traumatic emotional experience, then persistently meditating on an overwhelmingly traumatic experience while practicing inaction produces just those conditions. In effect, it could act like a kind of psychological self-torture. Meditation, in this case, would begin to lead to a perceived loss of allostatic self-efficacy, rather than toward the liberating states of self-understanding it is meant to.

## Author Contributions

All authors provided equal contributions to all sections, and so names appear in alphabetical order.

## Conflict of Interest

The authors declare that the research was conducted in the absence of any commercial or financial relationships that could be construed as a potential conflict of interest.

## Footnotes

1 See Ciaunica et al. (2020) for a recent phenomenological account of depersonalization and meditative insight.

2 For an excellent account of the neuroscience supporting the role of affect (including valence and arousal) in simultaneously tracking the relationship between the organism and the environment, and preparing the organism to make improvements to that relationship, see Lisa Feldman-Barrett’s work (2017).

3 Thanks to Lars Sandved-Smith for discussions about the computational nature of depersonalization.

4 Interestingly, this account is suggestive that the hyperreflective tendency to check one’s body and one’s current state common in people suffering from depersonalization (Colombetti and Ratcliffe, 2012) could be understood as compensatory behavior aimed at reducing the uncertainty relating to the perceived loss of control over interoceptive states.

5 For a neuroscientific account of this relationship between identification and motivation, see Damasio (2003).

6 See Smith et al. (2020) for a computational model of this process.

7 Notice here that the common phenomenology of long-term meditators being able to simply “let go” of mental and emotional distractions is closely related to the *increase* in control that we are proposing this meditative process engenders.

8 See Lindahl and Britton (2019) for reasons why that might be challenging.

9 See also Segal (2002) and Lindahl and Britton (2019) for clear accounts of the relationship between meditation and depersonalization.
